# New Insights into the Development and Morphogenesis of the Cardiac Purkinje Fiber Network: Linking Architecture and Function

**DOI:** 10.3390/jcdd8080095

**Published:** 2021-08-07

**Authors:** Caroline Choquet, Lucie Boulgakoff, Robert G. Kelly, Lucile Miquerol

**Affiliations:** Aix-Marseille Université, CNRS UMR 7288, Developmental Biology Institute of Marseille, Campus de Luminy Case 907, CEDEX 9, 13288 Marseille, France; caroline.choquet@univ-amu.fr (C.C.); lucie.boulgakoff@univ-amu.fr (L.B.); robert.kelly@univ-amu.fr (R.G.K.)

**Keywords:** ventricular conduction system, Purkinje fiber network, cardiac morphogenesis, cardiac progenitors, conduction defects

## Abstract

The rapid propagation of electrical activity through the ventricular conduction system (VCS) controls spatiotemporal contraction of the ventricles. Cardiac conduction defects or arrhythmias in humans are often associated with mutations in key cardiac transcription factors that have been shown to play important roles in VCS morphogenesis in mice. Understanding of the mechanisms of VCS development is thus crucial to decipher the etiology of conduction disturbances in adults. During embryogenesis, the VCS, consisting of the His bundle, bundle branches, and the distal Purkinje network, originates from two independent progenitor populations in the primary ring and the ventricular trabeculae. Differentiation into fast-conducting cardiomyocytes occurs progressively as ventricles develop to form a unique electrical pathway at late fetal stages. The objectives of this review are to highlight the structure–function relationship between VCS morphogenesis and conduction defects and to discuss recent data on the origin and development of the VCS with a focus on the distal Purkinje fiber network.

## 1. Introduction

**Figure 1:**
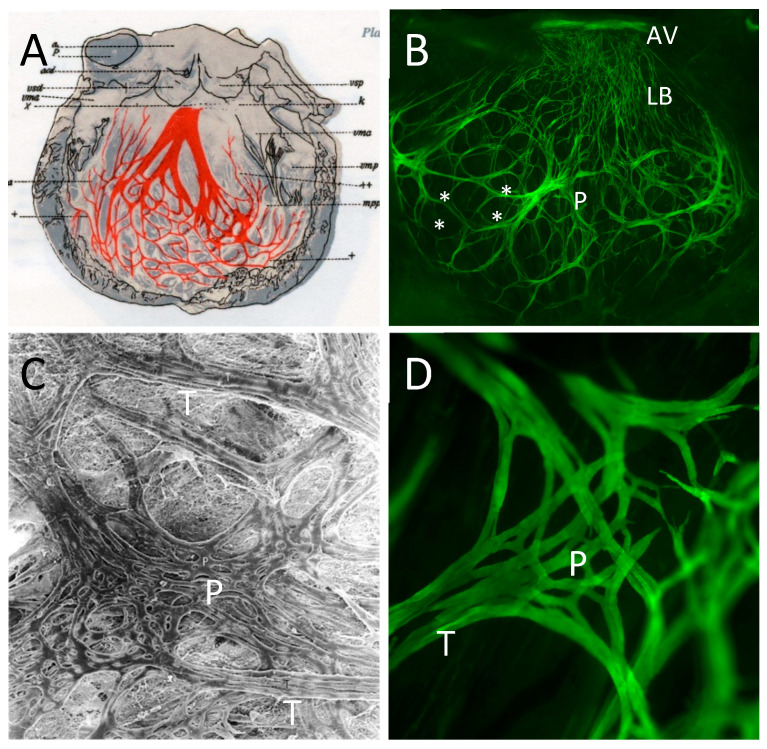
Morphological and cytoarchitectural similarities between human and mouse left ventricular Purkinje network. (**A**) Drawing of the human left ventricular conduction system from Tawara [1]. (**B**) Fluorescent image showing the mouse left ventricular conduction system of a *Cx40-GFP* heart [13]; The complexity of the PF network formed by ellipsoidal structures are indicated by stars. AVB: Atrioventricular bundle; LBB: Left Bundle branch; PF: Purkinje fibers. (**C**) Purkinje network (P) and muscular trabeculae (T) in the human heart. Purkinje cells running in parallel within the trabeculae are continuous with a delicate network of polygonal or stellate cells ×120 (a gift from Dr Shimada with permission) [14]. (**D**) Cytoarchitecture of the mouse Purkinje network from a high-magnification image of a *Cx40-GFP* heart shows similar organization with parallel fascicles (T) and Purkinje network (P) ×80.

Cardiac Purkinje fibers (PF) are named after the famous Czech physiologist Jean Evangelista Purkinje who first described “large gray gelatinous fibers” in the sheep heart in 1845 [1,2]. These cells were later described as specialized cardiomyocytes characterized by distinct histological features with pale cytoplasm, few mitochondria, a large amount of collagen, and a low number of myofibrils [3,4]. At the beginning of the 20th century, the description of the “stimulus conduction system” as a tree-like structure by Suneo Tawara was a major advance in our understanding of the link between cardiac structure and function [5]. The cardiac conduction system (CS) generates and propagates electrical activity through the heart to synchronize the consecutive contractions of the atria and ventricles [6]. Electrical activity originates in the pacemaker cells of the sinus node in the right atrium and is relayed to the atrioventricular (AV) node before being transmitted to the ventricles by a well-defined pathway known as the ventricular conduction system (VCS). The proximal part of the VCS, the His or AV bundle, emerges from the AV node, crosses the fibrous AV junction, and divides into the left and right bundle branches (BB). The distal portion of the VCS is composed of ramifications of BB into a complex network of PF fascicles forming ellipsoidal structures in each ventricle [7] (Figure 1). The VCS is characterized by fast conduction properties and automaticity through the expression of the high conductance gap junction Connexin 40 (*Cx40*; *Gja5* gene) and specific ionic channels [3,8]. This fast-conducting pathway is necessary to activate ventricular myocardium from the apex to the base, ensuring efficient expulsion of the blood through the great arteries. While these cells represent a tiny fraction of the ventricular volume (1–2%) [9], their pathogenic role is disproportionately high given that they are thought to cause ectopic activation that can lead to life-threatening ventricular arrhythmias in structurally normal hearts and particularly in patients with cardiac disease [10]. In ventricular arrhythmia, Purkinje cells generate both automatic and triggered focal rhythms, while the architecture of the PF network favors re-entrant circuits, sustaining ventricular tachycardia and fibrillation [11,12].

Modeling of the cardiac conduction system demonstrates a precise alignment of activation maps and PF network anatomy, illustrating how morphology underlies function [15,16,17]. Acquiring more accurate modeling of conduction disorders requires detailed imaging of the PF network [18,19]. However, this task is very challenging because it is not currently possible to capture the intact three-dimensional Purkinje network structure including Purkinje–myocardial junctions (PMJ) [20]. This complexity extends to architectural and anatomical differences in the PF network among mammalian species [14,18]. The PF network is uniquely present in mammalian and avian ventricles as a specific evolutionary trait necessary for the rapid heart rate of endothermic vertebrates [21]. It is generally localized at the subendocardial surfaces of the ventricles, as, for example, in human and rodents, while in avian, ungulate, and cetacean hearts, such as chick, sheep, pig, or whale, numerous penetrating fibers also invad the myocardial wall [22]. Other differences such as PF network mesh size or the size and shape of PF cells have been described and may reflect differences in conducting properties observed between species [14]. In the last two decades, through the development of new genetic tools, several groups have been able to explore the morphology of the ventricular conduction system in the mouse. Indeed, a specific staining or labeling by a reporter gene is required to fully appreciate the complex anatomy of VCS network [13,23,24,25]. In mice, the architectur of the PF network is highly similar to that of the human heart. In both species, the cytoarchitecture of the ellipsoidal fascicles comprises strands of elongated Purkinje cells which are joined by a delicate network of stellate cells [13,14,26] (Figure 1). The cardiac PF network presents a fascinating and intriguing architecture, and understanding its development and morphogenesis represents a real challenge but one that is essential to provide new insights into the origins of ventricular arrhythmias. Important results concerning the developmental origin and genetic mechanisms involved in PF network morphogenesis have been obtained recently using the mouse model and are presented in this review.

## 2. Developmental Origin of the Purkinje Fiber Network

While a better understanding of the origin and specification of the PF network is essential to comprehend the etiology of ventricular arrhythmias, the low proportion of PF cells within the adult heart and the lack of specific developmental PF markers render this analysis challenging. The permanent labeling of progenitor cells using clonal analysis and genetic tracing has been successful in determining from which progenitor cells the conductive cells originate during embryogenesis and when specification toward the PF lineage takes place.

### 2.1. Clonal and Genetic Tracing Analyses Reveal the Myogenic Origin of the VCS

Conductive cells were once thought to have a neuronal origin because of certain similarities with neurons such as morphology, electrophysiology, and gene expression. This idea was further reinforced as the localization or ablation of neural crest cells in the developing heart strongly suggested that this population may participate in the development of the proximal VCS [27,28]. However, genetic tracing of neural crest derivatives in chick [29] or in mice [30] excludes any contribution of this lineage to the VCS. An important finding came from clonal studies in chick which unambiguously showed a myogenic origin of the VCS. Indeed, retroviral labeling of single myogenic progenitors results in derivative cells forming clones composed of both PF cells and contractile cardiomyocytes [31]. Similar results were obtained in mice where a defective *nlaacZ* reporter gene under the control of the cardiac specific *α-cardiac actin* promoter was used to generate rare spontaneous β-galactosidase-labeled clones. This retrospective clonal analysis revealed the existence of mixed clones (composed of both conductive and contractile cells) and unmixed conductive clones (exclusively composed of conductive cells) [9]. The presence of large clones covering the different compartments of the VCS including AVB, BB, and PF argues in favor of a very early common progenitor [30]. No clones were observed containing cells covering both proximal (AVN-AVB) and distal (PF) VCS components, reflecting the independent origins of these compartments [29]. Genetic lineage analysis has demonstrated that the AVN derives from the atrioventricular canal while the AVB originates from ventricular cardiomyocytes [32]. The presence of mixed clones demonstrated that conductive cells from all CCS components share a common myogenic cardiac progenitor with neighboring contractile cells [9,33]. The low number of cells per unmixed conductive clone revealed that the proliferation rate decreases after restriction to the conductive fate. Indeed, cell cycle exit has been demonstrated to be an early indicator of conduction system lineage specification [34,35]. In the mouse, a genetic lineage study of mesodermal derivatives has showed that about 80% of conductive cells expressing the *CCS-lacZ* transgene were labelled in *Mesp1*-derived cardiomyocytes demonstrating further evidence for the myogenic origin of VCS [36]. However, this study also highlights that a small part of the VCS may arise from another embryonic layer, raising unresolved questions about their origin. Together these results demonstrated for the first time that contractile and conductive cells originate from the same myogenic progenitors, excluding a neuronal origin of the VCS, that proximal and distal conductive cells originate from different lineages and that segregation of VCS cells is followed by limited outgrowth. 

The heart is formed from two populations of cardiac progenitors, the first (FHF) and second heart fields (SHF) which emerge sequentially in nascent mesoderm [37,38]. Additional lineage tracing experiments have demonstrated the dual contribution of the FHF and SHF myogenic progenitor cells to the VCS [30,39,40]. The left BB and left PF network derive exclusively from the FHF lineage whereas the right PF network and SAN derive exclusively from the SHF lineage [41]. On the other hand, AVN, AVB, and right BB, which develop at the level of the interventricular septum where the two cardiac fields meet, derive from progenitors of both lineages. These data demonstrate that conductive cells do not derive from an independent lineage but rather that development of the VCS follows that of heart morphogenesis.

### 2.2. A model of VCS Morphogenesis Based on the Expression of Conductive Markers

Despite the fact that ventricular conductive cells form a unique fast-conductive pathway in the adult heart, none of the specific markers identified so far are exclusively expressed in these cells which would have been very helpful to draw a precise picture of VCS morphogenesis. This implies that cardiac progenitors segregate into different lineages as differentiation occurs. During development, several markers delineate a population of cardiomyocytes forming a ring at the interventricular foramen at E9.5 in the mouse embryo [42,43]. The *CCS-LacZ* transgene has been described to label the CCS and is also present in the interventricular septum and valves [24]. *Tbx3* is expressed in the proximal VCS, including AVN, AVB, and BB, but not in distal PF cells [44]. These two markers identify a population of cardiac progenitors previously described as the primary ring and thought to label proximal VCS precursors [45]. At early stages of embryonic development ventricular activation colocalizes with the primary ring [46].

In contrast, prospective clonal analysis of *Cx40* expressing cells shows that the distal VCS including PF cells originates from ventricular trabeculae [9]. Indeed, ventricular trabeculae expressing the gap junction protein Cx40 constitute the primary fast-conductive pathway during embryogenesis [47]. *Cx40*+ genetic tracing analysis demonstrated that PF cells share a common progenitor with contractile myocytes localized within ventricular trabeculae [48]. Progressive lineage restriction to a conductive phenotype during development is largely complete by E16.5 and followed by limited proliferative outgrowth [9]. These data have been confirmed by genetic tracing analysis using the semaphorin 3A (*Sema3A*) gene to specifically label embryonic trabeculae [49]. As trabecular cells differentiate into PF cells, the ventricular activation pattern switches from the primary ring to an apex-to-base pathway [46]. These data suggest progressive acquisition of a functional VCS during ventricular morphogenesis. This model is supported by the gradual spatiotemporal restriction of the expression of conduction-specific genes during ventricular myocardial growth controlled by a complex transcriptional regulatory network [50,51]. Most PF markers analyzed to date are expressed in surrounding contractile tissue prior to their restriction to conductive cells including *Cx40*, *Irx3*, *Etv1*, and *Sema3a* which are expressed in ventricular trabeculae before being progressively restricted to the mature VCS [13,52,53,54]. Consistent with this model, the chick VCS has been shown to develop by a process of induction and recruitment (ingrowth model) of localized cardiomyocytes through endothelial derived signals, i.e., endothelin, explaining in part why the distal avian VCS, unlike in the mouse, develops next to coronary arteries [55,56,57]. In contrast to chick, specification of the murine cardiac conduction occurs in the absence of endothelin signaling [58,59].

Other markers switch on during the progressive differentiation and physiological maturation of conductive cells. This is the case for *Cx40* in the AVB and BB at fetal stages, where it is essential for fast conduction [60]. During embryogenesis, HCN4 (hyperpolarization-activated cation-selective nucleotide-gated channel 4) expression also delineates the atrioventricular ring [61] and HCN4 is re-expressed during fetal stages (E16.5) in the entire CCS where it mediates the pacemaker function of conductive cells [39]. The axonal glycoprotein Contactin-2 (Cntn2) is only expressed in the mature VCS from perinatal stages [25,51]. Recent single-cell transcriptomic analysis of CCS components at E16.5 has identified numerous additional genes expressed within different CCS components. Many of the genes expressed in the PF cluster are also expressed within other CCS clusters [62].

### 2.3. Identification of Conductive Progenitors and Early Commitment of Purkinje Fibers

In order to investigate the time at which early cardiac progenitors become restricted to the conductive lineage during development, temporal clonal analyses were performed. The multicolored R26-confetti reporter line was been crossed with a tamoxifen inducible *Cre* line under control of the *Smooth Muscle Actin* (*SMA*) promoter to individually label early cardiac progenitors at the cardiac crescent stage [63]. The observation of unicolor clones exclusively in the AVB demonstrates the early segregation of cells towards a conductive fate in this early cardiac progenitor population. Segregation towards the BB and PF lineages arises later during development as non-conductive cells that are clonally related to cells within these structures are also present in the surrounding septum. A population of cardiac progenitors committed toward the conductive lineage thus exists at the linear heart tube stage. In addition, a temporal genetic tracing of embryonic *Tbx3*+ cardiomyocytes reveals the presence of AVB-committed cells in the outflow tract of the linear heart tube and the establishment of the AVB by progressive fate restriction of *Tbx3*+ cardiomyocytes [64]. However, these *Tbx3*+ progenitors, representative of the primary ring, do not contribute to the PF lineage [64]. More recently, clonal analysis has identified, for the first time, a subpopulation of early cardiac progenitors committed to the PF lineage as early as the onset of heart tube formation (E7.75), prior to the onset of trabeculation [65]. Labeling early SMA+ cardiac progenitors at around E7.5 has been previously shown to mark derivatives of FHF, confirming a FHF origin of the left ventricular PF network. The early commitment of conductive cardiomyocytes has been proposed to constitute a “scaffold” for subsequent growth of the PF network [65]. Temporal clonal analysis of single trabecular cells demonstrated that committed PF cells are already present at the onset of trabeculation and their proportion increases as the ventricle grows [65].

Thus, the VCS develops from two independent populations of early committed precursors in the form of the primary ring and ventricular trabeculae in the embryonic heart that will give rise respectively to the proximal or distal components of the VCS. At fetal stages, all cells of the VCS acquire a fast-conductive phenotype to form an integrated functional ventricular electrical pathway, induced by the spatiotemporal control of complex transcriptional mechanisms [50,66,67]. This is supported by optical mapping experiments revealing that ventricular conduction switches from a single activation at the level of the Primary ring to dual activation in both ventricles along an apex to base axis [46]. Future single-cell transcriptomic analyses at different embryonic timepoints will be necessary to more fully dissect the cellular heterogeneity of ventricular trabeculae and the molecular landscape of these subcomponents of the VCS. 

## 3. Defective VCS Morphogenesis Causes Conduction Defects in Mouse Models

### 3.1. Structure–Function Relationship between VCS Architecture and Cardiac Conduction

Ventricular conduction defects, including bundle branch blocks, represent unfavorable prognostic signs linked with morbidity and mortality and are often associated with abnormal Purkinje cell biology [68]. However, the role of the Purkinje network morphology in the genesis and maintenance of certain types of conduction defects is not completely understood. The rapid propagation of electrical activity in the ventricles results from two main parameters, namely, conduction velocity and the architecture of the PF network, defects in either of which represent a major substrate for ventricular arrhythmias [4]. Using mouse genetic models, a striking correspondence has been revealed between VCS morphology and electrical activation indicating a strong structure–function relationship [13,69]. The *CCS-LacZ* mouse line expresses a beta-galactosidase encoding reporter gene in the developing VCS and its pattern of expression is correlated with the conduction pathway detected by optical mapping in embryonic heart [24,70]. In the adult mouse heart, the right and left BBs are morphologically and functionally asymmetric as depicted by *Cx40* expression in knock-in *Cx40^GFP/+^* mice [13,71]. Whole-mount fluorescent and optical mapping images of each side of the septum show a unique thin right BB compared to a large left BB composed of multiple strands. The PF network is also asymmetrical between ventricles with ramifications mainly present at the surface of the right ventricular free wall while the PF covers the entire septal surface in the left ventricle [7,9]. Detailed analysis of PF distribution in the LV indicates a peak of fibers about one-third of ventricular length above the apex at the location where cell–cell communications through Cx43 junctions are seen between PF and working myocardium suggesting a high density of PMJ in the distal part of the PF network [72]. Three-dimensional mathematical models of heart activation based on the asymmetrical distribution of the PF network in both ventricles match with experimentally recorded activation maps [16,17,72]. The asymmetrical localization of the dense network of Purkinje fibers between the right and left ventricles is necessary to optimally synchronize contractions according to the blood flow pattern of each ventricle [7].

Until recently, conduction defects have been mainly attributed to reduced conduction velocity or abnormal electrophysiological properties of cardiomyocytes [66], and the role of VCS morphology was poorly appreciated. However, advances in deciphering the molecular regulatory network responsible for PF specification and function have improved our understanding of their contribution to conduction defects [67,73]. In 2004, two studies described for the first time that abnormal VCS structure is associated with conduction defects [74,75]. Since then, several genes have been implicated in VCS morphogenesis, and their deletion results in either a reduction or an increase of conductive cells which impacts on both VCS architecture and function. 

### 3.2. Mouse Models with a Hypoplastic VCS

Neuregulin1 (Nrg1) is a paracrine factor and a member of the EGF family implicated in the regulation of growth and differentiation of different cell types [76]. During cardiac development, endocardial-derived Nrg1 is the initiating factor of trabeculae formation [77] and is sufficient to induce a massive conversion of contractile cardiomyocytes towards a conductive phenotype in organ culture of embryonic mouse hearts [70]. Downstream of Nrg1, the Ras/MAPK pathway induces the activation of ETV1, a transcriptional activator, member of the Pea3 group of ETS family transcription factors [52]. Similarly to Nrg1, ETV1 is sufficient to induce a conductive fate in vitro in rat neonatal ventricular myocytes and human induced pluripotent stem cell-derived cardiomyocytes [52]. Mice lacking *Etv1* develops a highly hypoplastic His–Purkinje network in which ventricular conduction is affected, as measured by an increased QRS duration.

Like ETV1, transcription factors such as Nkx2-5, Tbx5, and Irx3 are fundamental regulators of VCS specification and maturation and act synergistically in those processes [78]. In consequence, hemizygous mutation of one or several of these genes leads to defects in VCS morphology characterized by hypoplasia of one or multiple parts of the conduction system.

*Nkx2-5* encodes a cardiac transcription factor that regulates major molecular pathways involved in heart development and function, with a particular role in the morphogenesis of the conduction system. *NKX2-5* mutations have been described in many patients with congenital heart defects and the majority of them present conduction defects or arrhythmias [79,80,81]. *NKX2-5* has been mainly found to be involved in atrial arrhythmias and AV block, however, a long-term follow-up revealed a high incidence of sudden cardiac death (SCD) on aging in these patients suggesting the occurrence of ventricular arrhythmias [82]. In mice, *Nkx2-5* haploinsufficiency does not lead to major structural abnormalities of the heart, except for very rare ASD [83]. However, mice lacking one copy of *Nkx2-5* systematically develop hypoplasia of both the proximal and distal VCS [84]. Consistent with a smaller AVN, *Nkx2-5* heterozygote mice develop atrioventricular conduction delays with age as measured by an increased PR interval from P7 [85,86,87]. The PF network architecture of these mice is also greatly affected, especially in the apex, as they show a 2–3-fold decrease in conductive cells numbers, together with a decrease in the number of the ellipsoidal structures that characterize the Purkinje network [84]. As a consequence, decreased conduction velocity in the ventricles is detected in *Nkx2-5* haploinsufficient hearts, together with a prolonged QRS [84,87]. The recording of a normal action potential in *Nkx2-5* haploinsufficient isolated PFs suggest that conduction defects are directly related with PF network hypoplasia rather than a cellular dysfunction [84]. 

*Tbx5* is a member of the family of T-box transcription factor-encoding genes. During embryonic mouse development, *Tbx5* is expressed in left ventricular myocardium and a small portion of the right ventricle [88,89]. As development proceeds *Tbx5*, like *Nkx2-5*, is transcribed at higher levels in the VCS than in the surrounding myocardium [75]. In human, *TBX5* haploinsufficient mutations cause Holt–Oram syndrome characterized by upper limb defects, heart defects, and conduction disturbances including AV and BB blocks [90]. Similarly, haploinsufficient mice for *Tbx5* (*Tbx5*^+/−^) fail to express the atrial natriuretic factor ANF (Nppa) and Cx40 [91]. They also present a failure of AVB and BB maturation manifested as a foreshortened AVB, large and immature LBB and hypoplastic, if not absent, RBB [75]. Reduced gap junction expression together with morphological defects result in the adult in prolonged atrioventricular (long PQ interval) and ventricular (long QRS) conduction [75]. Together, this suggests a critical role of Tbx5 in the specification and function of the proximal VCS. 

Compound *Nkx2.5^+/−^::Tbx5^+/−^* heterozygous mice have also been generated [34] and possess increased conduction system defects compared to the single heterozygotes. Their VCS morphology is greatly affected as neither *minK^LacZ^* nor *Cx40* expression can be observed in the AVB and BB. Consequently, ventricular conduction is more severely slowed in double *Nkx2.5^+/−^::Tbx5^+/−^* heterozygous mice compared to single mutants, as measured by QRS duration in neonatal mice.

Irx3 is a member of the Iroquois transcription factor family that has been described to have a dual action on the maturation of the VCS. During embryonic development, Irx3 acts synergistically with Tbx5 and Nkx2.5 to upregulate the expression of *Cx40* in the developing VCS while downregulating the expression of *Cx43* [54]. At postnatal stages, Irx3 is also critical for the postnatal maturation of the VCS, as it plays a key role in cell cycle exit and differentiation of the conductive progenitors [78]. As a consequence, *Irx3*^−/−^ mice possess a hypoplastic VCS characterized by slow ventricular conduction (long QRS) and frequent right bundle branch block. Cellular conductance might also be affected in *Irx3*^−/−^ hearts as Cx40 content is reduced by 2-fold compared to controls. As conduction in *Cx40* heterozygous mutant mice is normal, many of the conduction defects seen in *Irx3*^−/−^ mice cannot be solely explained by reduced *Cx40* expression. Thus, the defective architecture of the VCS in *Irx3*^−/−^ mice is likely to play an important role in the development of cardiac conduction defects [78]. Ventricular tachycardia is also frequent in *Irx3*^−/−^ mice [92]. However, whether this ventricular arrhythmia originates from decreased conduction protein levels or hypoplasia of the VCS has not been determined.

Thus, to date, Tbx5 is known to be critical for the proper maturation of the AVN and proximal VCS mostly, Nkx2-5 is required for the specification of all components of the VCS, while Irx3 regulates all components of the VCS development. This suggests that the maturation of each component of the conduction system is controlled by unique gene regulatory networks.

Altered expression of downstream effectors of these transcription factors could also lead to hypoplastic architecture of the conductive system. This is the case for miR1 and Id2 for instance, which regulate conductive fate specification and/or conductive maturation. 

miR1 is the most abundant cardiac microRNA and is a direct transcriptional target of Nkx2-5 [93,94]. In mice, cardiomyocyte-driven overexpression of miR1 results in VCS hypoplasia arising at E13.5 and persisting in adulthood [95]. Indeed, premature upregulation of miR-1 during embryogenesis inhibits proliferation of PF precursors, possibly through downregulation of the cyclin dependent kinase Cdk6 which by consequence results in an upregulation of the pocket proteins. Overexpression of miR1 also perturbed Ca2+ handling in PF via its inhibiting effects on the SNARE protein Syntaxin 6 (Stx6), involved in cellular trafficking [96]. Thus, miR1 overexpressing mice present various conductive defects such as tachycardia, arrhythmia, slow atrioventricular conduction (long PR) or AVB, and slow ventricular conduction (long QRS) [97]. Some of those defects, including defective ventricular conduction, could be attributed to VCS morphological defects.

Id2, a member of the ID gene family of helix-loop-helix-containing transcription repressors, is particularly enriched in the AVB and BB at fetal stages and is regulated both by Nkx2-5 and Tbx5. In the conduction system, Id2 represses the activity of myogenic factors such as MyoD [98], thus favoring a conductive fate. Similar to *Tbx5*^−/−^ mice, *Id2*^−/−^ mice possess an immature-like AVB and LBB characterized by an absence of well-defined His bundle and a broad LBB originating from the entire length of the ventricular crest, although morphology of the PF has not been investigated in this mutant so far [34]. Consistent with the defective proximal VCS morphology, *Id2*^−/−^ mice display conduction defects in the BB, resulting in an increased QRS duration and frequent fragmented QRS complex as described by RsR’ pattern on ECG indicative of LBB block.

Neural cell adhesion molecule-1 (NCAM-1) is an adhesion molecule of the immunoglobulin superfamily (IgSF-CAMs). In addition to mediating cell–cell interaction, NCAM-1 binding triggers activation of downstream signaling cascades, which have been extensively studied in the context of neural development. In Ncam-1 KO mice, the expression of several PF specific genes is downregulated [99]. Among them, some are involved in cellular conductance—such as *Cx40* and Scn4b—whereas others, like ETV1, are necessary for conductive cell specification. The subcellular localization of NCAM-1 is regulated by its polysialic acid post-translational modification, and it is necessary for the localization of *Cx40*, Cntn2, and Scn4b at the level of the intercalated discs. Moreover, *NCAM-1* KO mice display an 30% hypoplasia of the VCS, specifically in the mid and apical LV. Functionally, ventricular conduction velocity is decrease in these mice, as shown by an increased QRS duration. However, the relative contribution of altered cellular conductance and structural defects of distal VCS to this phenotype was not compared.

Other downstream effectors of Nkx2-5, Tbx5, and Irx3 only act through regulation of the expression of conduction proteins without impacting on VCS architecture. Hopx, a homeobox transcription factor, is one example as it has been demonstrated to be required for proper *Cx40* expression in the VCS downstream to Nkx2-5 [100]. Knock-out mutation of such downstream effectors leads to conduction defects, such as QRS elongation, without causing architectural defects. 

**Figure 2:**
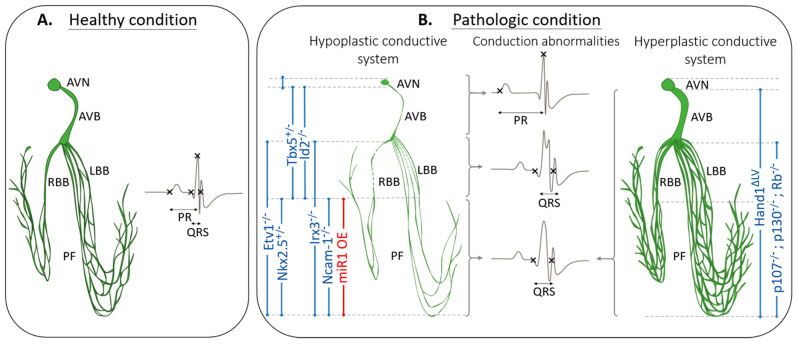
Correlation between structural defects of specific components of the VCS and conduction abnormalities. (**A**) Representation of the ventricular conduction system of healthy mice, with a corresponding surface ECG. (**B**) Representation of morphological abnormalities of one or several components of the ventricular conduction system with the corresponding mouse genotype indicated vertically. Abnormal ECGs are indicated with an increased PR interval associated with hypoplasia of the AVN and/or AVB; a fragmented QRS also referred as RsR’ pattern associated with hypoplasia of bundle branches and a prolonged QRS duration associated with both hypoplasia and hyperplasia of the Purkinje network. AVN: Atrioventricular node, AVB: Atrioventricular bundle, RBB and LBB: right and left bundle branch, PF: Purkinje Fibers, OE: overexpression, ΔLV: mutation of the left ventricle specific enhancer.

Taken together, these studies support the notion that hypoplasia of a part of the conduction system is responsible for slowing down conduction in the corresponding VCS compartment. Indeed, a hypoplastic AVB is characterized by slow atrioventricular conduction, resulting in an increased PR duration. Similarly, a hypoplastic Purkinje network shows slow ventricular conduction, and thus, a prolonged QRS interval (Figure 2).

### 3.3. Mouse Models with Hyperplastic VCS

Increase of progenitors committed toward a conductive fate, and/or perturbed proliferation of conductive progenitors, could lead to an excessive ventricular conduction system. However, few hyperplastic models have been described to date (Figure 2).

The transcription factor Hand1 is expressed in the VCS in the adult heart [101]. Downregulation of *Hand1* by deletion of a left ventricular specific enhancer in transgenic mice provokes morphologically abnormal development of the VCS. In contrast to the PF hypoplasia seen in *Nkx2-5*^+/−^ mice, *Hand1* mutant hearts display a dysmorphic and hyperplastic VCS [101]. Interestingly, an excessive VCS in this model is also associated with reduced conduction velocity in the ventricles (long QRS and perturbed activation map upon atrial pacing).

Pocket proteins p107, p130, and Rb are key regulators of the cell cycle exit in many cell types. They are expressed in the myocardium during development—for p107 and p130—and during the neonatal and adult periods for Rb [102]. The impairment of their activity in *p107*^−/−^, *p130*^−/−^, or α-*MHC^Cre^::Rb^fl/fl^* mice perturbs cell cycle exit of conductive progenitors, leading to hyperplasia of the developing VCS. However, the phenotype is lethal during late fetal stages making functional study impossible.

These examples illustrate that while morphological defects are invariably associated with conduction defects, the opposite is not true. Malfunction and/or malformation of the Purkinje network can lead to conduction delay, dyssynchronous contraction, and tachyarrhythmias, and these are important sources of morbidity in patients with congenital and acquired heart disease [4,10]. However, classification as a function of their etiology would be valuable in terms of developing preventive or curative therapeutic strategies in patients. Up to now, no morphological defects of the PF network have been reported in human patients. The main limitation of these studies is that not all of these genes are specific to the conduction system during development and defects in other non-conductive cardiomyocytes could impact cardiac function in these models. In addition, many of these mice do not survive after birth, making it impossible to analyze the mature morphology of the adult conduction system. To address these problems and to understand the intrinsic role of each of these genes on the development of the ventricular conduction system as well as their genetic interactions, conditional loss of function of these genes specifically in the different subcomponents of the VCS should be performed in the future. However, while the regulatory determinants of this developmental program have not been fully established, a key factor in determining the functional properties of the whole unit is the maintenance of the appropriate ratio of conductive to working cardiomyocytes: PF hypoplasia leads to conduction defects and ventricular tachycardia [74,78] while PF excess causes reduced contractile force and conduction defects [102].

## 4. A Two-Step Model of PF Network Morphogenesis Involving Scaffold and Recruitment Phases

The development of the VCS is far from linear, as proposed by the primary ring model with a committed progenitor population that expands with the growth of the interventricular septum (outgrowth model) [45]. As described above, the progressive restriction of the conductive lineage during ventricular development highlights an alternative model with a gradual development of the different components of the VCS resulting in a unique and functional electrical pathway [51]. The development of the PF network appears to be intimately linked to ventricular wall morphogenesis and trabecular cardiomyocytes in particular [103]. Thus, the *Cx40-CreERT2* mouse line which targets ventricular trabeculae and VCS represents an invaluable tool to investigate the relationship between trabecular cell fate and the development of the VCS. Temporal genetic tracing of single trabecular cells has shown to be a powerful approach to decipher PF morphogenesis by comparing their cellular fate and behavior in control and in *Nkx2-5*^+/−^ mice [65].

### 4.1. The PF Network Grows by Two Phases of Recruitment

**Figure 3:**
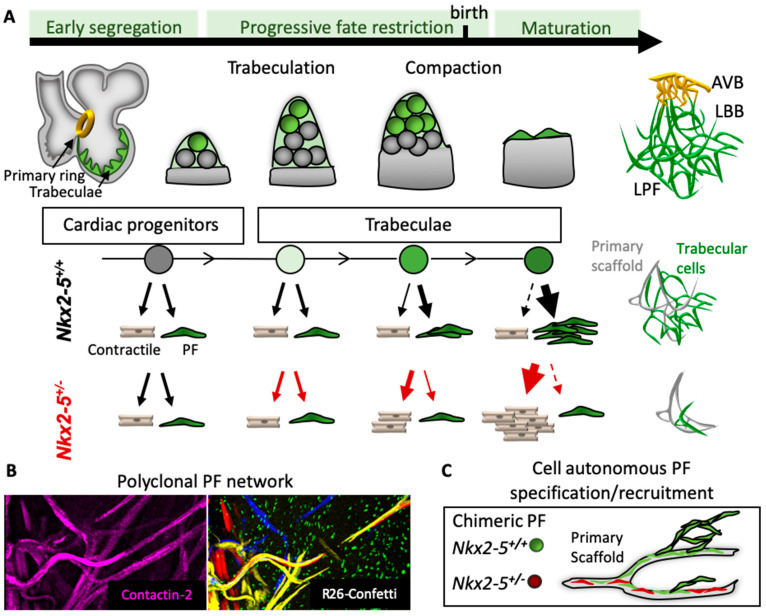
Model of the ventricular conduction system lineage and morphogenesis. (**A**) Within the early heart tube, a subset of cardiac progenitors localized in the primary ring or emerging trabeculae is already specified to the conductive lineage (green cells) and represents the primary scaffold of the VCS network. During trabeculation and compaction of the ventricles, trabecular progenitors (gray cells) are progressively fate-restricted, and many conductive cells are recruited to the primary scaffold to build a complex PF network. At late fetal stage, trabecular cells enter the conductive lineage in a massive Nkx2-5-dependent recruitment to contribute to ellipsoidal structures of the PF network. In *Nkx2-5*^+/−^ mice, the conductive potency is progressively lost leading to a hypoplastic PF network with very rare ellipsoidal structures. (**B**) Whole-mount fluorescence images of the left PF network from *Mesp1-Cre::R26R-Confetti* mice at P21. Immunostaining for Contactin-2 (CNTN2) is used to label the mature PF network. Multicolor confetti cells show the polyclonal morphogenesis of the PF network. (**C**) Schematic representation of chimeric PF network where *Nkx2-5*^+/−^ cells (red) are not recruited nor recruiting and do not form ellipsoidal structures of the PF network unlike *Nkx2-5*^+/+^ cells (green) which cell autonomously participate to the PF network.

At early embryonic stages, a large proportion of trabecular cells are bipotent and give rise to conductive and contractile cardiomyocytes; however, this number decreases progressively during ventricular development and disappears at birth (Figure 3A). Committed conductive or contractile cells are already present at the onset of trabeculation and the proportion of conductive cells increases with time while remaining constant for contractile cells. The increase in proportion of committed PF is low until fetal stage and high around birth suggesting a late cell fate decision of many trabecular cells to enter the PF lineage. In *Nkx2-5* heterozygous mutant mice, the number of early committed PF is slightly reduced in comparison to control, while the commitment of new PF cells to the network at late fetal stage is almost completely abolished leading to a hypoplastic PF network. In each clone, the number of conductive cells is constant over time and in different genotypes, suggesting that the growth of the PF network does not result from proliferation of precursor cells but rather by the recruitment of newly committed PF from the pool of bipotent trabecular cardiomyocytes. In summary, PF network morphogenesis is highly polyclonal (Figure 3B) and occurs by an initial specification of committed conductive cells from early cardiac progenitors to form a scaffold, followed by a progressive recruitment of PF from bipotent trabecular cells in two phases. The first recruitment phase is slow and independent of Nkx2-5 while the second phase is rapid and sensitive to a reduced level of Nkx2-5 expression. 

### 4.2. Cell-Autonomous Specification and Recruitment of the PF Network

The exact mechanisms involved in the progressive recruitment of committed PF within trabecular myocardium are still not yet understood, although Nkx2-5-dosage is essential for their specification and late fetal recruitment. The reduced number of PF in the *Nkx2-5*^+/−^ mutants results from a failure of trabecular cells to differentiate into PF rather than a loss of PF cells [84]. Moreover, cardiac progenitor cells failed to develop proximal and distal parts of the VCS in the absence of Nkx2-5 suggesting that a minimal level of Nkx2-5 is essential for the specification of the conductive phenotype [104]. Chimeric hearts made from a mixed population of *Nkx2-5*^+/+^ and *Nkx2-5*^+/−^ cells show a similar contribution of these cells in trabecular progenitors while PF originate mainly from wild type cells demonstrating that Nkx2-5 is required cell-autonomously for PF differentiation [84]. However, the lack of ellipsoidal structures persists in these chimeric hearts demonstrating that Nkx2-5 wild type cells do not rescue the defect in late fetal recruitment in *Nkx2-5* mutant hearts [84]. These data suggest that Nkx2-5 mutant cells are not efficient to recruit new PF. These results are supported by the lack of development of a complex PF network after conditional deletion of one *Nkx2-5* allele in embryonic ventricular trabeculae [104]. Conditional deletion of one *Nkx2-5* allele at birth using *Cx40-CreERT2* mice does not affect the architecture of the PF network or the differentiation of trabecular cells to PF [65]. However, if one allele of *Nkx2-5* is deleted during trabecular development, the PF network is severely affected. Thus, Nkx2-5-dosage is required for the morphogenesis of the PF network during embryonic and fetal development and not at postnatal stages. Together, these data suggest that the morphogenesis of the PF network complexity relies on a cell-autonomous recruitment to the primary scaffold.

### 4.3. PF Network Complexity Is Linked to Ventricular Trabeculae Compaction

The PF network is formed by complex ellipsoidal structures that are reminiscent of the morphology of embryonic ventricular trabeculae [105]. Our clonal analyses suggest that the formation of complex ellipsoidal PF structures is independent from trabecular cell fate but rather the consequence of ventricular myocardium maturation. Indeed, at late fetal stages, the trabecular myocardium coalesces to form a smooth ventricular wall by a process designed as compaction [106]. The conditional deletion of *Nkx2-5* in ventricular or trabecular myocardium leads to the development of an hypertrabeculated myocardium, showing that Nkx2-5 plays an important role in trabecular development and not only in PF differentiation [86,104]. The association between the formation of the PF network and trabecular compaction may explain differences of PF localization between the chick and the mouse. The presence of deep PF in the chick would originate from extensive trabecular compaction in comparison to the mouse in which trabecular compaction is restricted to subendocardial cardiomyocytes [107,108,109]. PF network morphogenesis is thus integrally related with ventricular maturation potentially explaining conduction defects associated with congenital malformations or inherited cardiomyopathies. A recent genetic association study has shown that the trabecular morphology in human is important for cardiac performance and found a causal relationship between trabecular morphogenesis and risk of cardiovascular diseases [110]. Interestingly, Meyer et al. identified 16 genes associated with the fractal structure of the heart that may be new players in shaping VCS architecture. Note that one of these genes, *HAND1*, has been recently implicated in VCS morphogenesis as shown by an excessive VCS after deletion of *Hand1* enhancer in mice [101]. Together, this suggests that slight dysregulation of the gene regulatory network controlling ventricular compaction during development may unveil a predisposition to cardiac dysfunction in the adult.

## 5. Conclusions

The VCS is a unique electrical circuit made of fast-conductive cardiomyocytes necessary to synchronize ventricular contractions. The architecture of the VCS is relatively complex and mirrors the pattern of ventricular activation. Conduction defects and ventricular arrhythmias are multifactorial disorders, and the functional importance of VCS architecture has recently emerged from studies of genetic models in mice. These models have revealed how anomalies in VCS morphogenesis during development contribute to conduction defects in adult mice. Thus, the structure–function relationship between VCS morphogenesis and electrical conduction is an important parameter to consider in cardiac conduction disease and ventricular arrhythmia. These mouse models have also provided insights into the embryonic origin and formation of the VCS and establish a model of PF network morphogenesis. The VCS derives from two pools of cardiomyogenic progenitors which will give rise separately to the AVB and BB from primary ring cells or to PF from ventricular trabeculae. Trabecular cells are progressively restricted to the conductive lineage starting at the linear heat tube stage until birth. The number of committed PFs increases by recruitment from trabecular cells in two phases. The scaffold phase is slow and independent of reduced levels of *Nkx2-5*, while during late fetal stages, a massive recruitment takes place, failure of which in *Nkx2-5*^+/−^ mice leads to PF network hypoplasia, highlighting how anomalies in VCS morphogenesis during development contribute to conduction defects in adult mice.

## Data Availability

Not applicable.
